# Kinship analyses identify fish dispersal events on a temperate coastline

**DOI:** 10.1098/rspb.2014.0556

**Published:** 2014-06-22

**Authors:** C. Schunter, M. Pascual, J. C. Garza, N. Raventos, E. Macpherson

**Affiliations:** 1 Centre d'Estudis Avançats de Blanes (CEAB-CSIC), Car. Acc. Cala St. Francesc 14, Blanes, Girona 17300, Spain; 2 Department of Genetics and IRBio, Universitat de Barcelona, Diagonal 643, Barcelona 08028, Spain; 3 Southwest Fisheries Science Center, National Marine Fisheries Service and University of California, 110 Shaffer Road, Santa Cruz 95060, USA

**Keywords:** parentage analysis, connectivity, dispersal, self-recruitment, sibship, *Tripterygion delaisi*

## Abstract

Connectivity is crucial for the persistence and resilience of marine species, the establishment of networks of marine protected areas and the delineation of fishery management units. In the marine environment, understanding connectivity is still a major challenge, due to the technical difficulties of tracking larvae. Recently, parentage analysis has provided a means to address this question effectively. To be effective, this method requires limited adult movement and extensive sampling of parents, which is often not possible for marine species. An alternative approach that is less sensitive to constraints in parental movement and sampling could be the reconstruction of sibships. Here, we directly measure connectivity and larval dispersal in a temperate marine ecosystem through both analytical approaches. We use data from 178 single nucleotide polymorphism markers to perform parentage and sibship reconstruction of the black-faced blenny (*Tripterygion delaisi*) from an open coastline in the Mediterranean Sea. Parentage analysis revealed a decrease in dispersal success in the focal area over 1 km distance and approximately 6.5% of the juveniles were identified as self-recruits. Sibship reconstruction analysis found that, in general, full siblings did not recruit together to the same location, and that the largest distance between recruitment locations was much higher (11.5 km) than found for parent–offspring pairs (1.2 km). Direct measurements of dispersal are essential to understanding connectivity patterns in different marine habitats, and show the degree of self-replenishment and sustainability of populations of marine organisms. We demonstrate that sibship reconstruction allows direct measurements of dispersal and family structure in marine species while being more easily applied in those species for which the collection of the parental population is difficult or unfeasible.

## 1. Introduction

Larval dispersal determines the connectivity patterns of many species of marine fish. Connectivity counteracts population structuring, and is crucial for the persistence and resilience of many species. This has been emphasized in conservation policies, including the design of networks of marine protected areas [1,2]. However, owing to the technical difficulties in tracking fish through the pelagic larval phase, direct measures of connectivity are scarce, and understanding the distribution of dispersal distances and their direction is still a great challenge in marine ecology [3].

One solution to overcome this problem is the use of parentage analysis to study dispersal in species with a relatively stationary adult phase [4]. Such analysis permits the estimation of connectivity, as the detection of parent–offspring pairs provides direct evidence of offspring dispersal, if adult movement patterns are known [5]. Since the first application of this method in the marine environment, a number of studies have been conducted that differ in scale and in study species, and they have revealed wide variation in self-recruitment (from approx. 7.5 to 64% [6,7]). While some studies have shown that larvae not recruiting back to the natal habitat ‘emigrate’, with dispersal success rapidly declining over short distances [8], others have found larval export from a marine reserve in the Great Barrier Reef to reach locations approximately 30 km away [9], and in Papua New Guinea up to 35 km in distance [4]. Even so, this could still be considered a small scale, especially for species with large geographical ranges or very discontinuous habitat.

The successful application of parentage analysis to the study of larval dispersal requires sampling of a sufficiently large proportion of the parental population for the successful encounter of parent–offspring pairs [10]. Owing to this, studies applying parentage analysis have been restricted to species with confined parental habitats [11,12] or that form reproductive aggregations [12]. However, few common, commercially harvested and endangered marine species, especially in temperate and cold water ecosystems, exhibit such characteristics. Other genetic approaches, such as reconstruction of sibling groups (sibships), might be useful to identify dispersal and connectivity patterns. Sibship reconstruction analysis is a common pedigree reconstruction method and has been used to study a variety of questions related to family structure and reproductive output in natural populations [13]. In the marine environment, where the high fecundity of most species increases the challenge of encountering kin, large full-sib families can be successfully identified and estimates of effective number of breeders derived [14]. In the terrestrial environment, studies using sibship analysis have focused on explaining whether siblings disperse together [15], but it is also possible to infer the range of the dispersal distances of a population using sibship reconstruction [16].

In the marine environment, the collection of samples from a large fraction of the population can be extremely difficult and time-consuming. Performing sibship analysis has the advantage that only one generation needs to be sampled, whereas parentage analysis requires sampling of both the adult and offspring generations. When extensive adult collection is not feasible, for example in species with high adult motility, sibship reconstruction can be an informative and suitable alternative approach for the estimation of dispersal.

In this study, we compare the usefulness of sibship and parentage analyses in deciphering patterns of dispersal and connectivity in the marine environment using a small rocky shore fish, *Tripterygion delaisi* (the black-faced blenny) [17], as a model system. It is one of the few temperate species that exhibits the necessary characteristics for parentage analysis and therefore allows direct comparison of both methodologies. We use data from 192 single nucleotide polymorphisms (SNPs) to perform parentage and sibship reconstruction analyses in the black-faced blenny on an open coastline of the northwestern Mediterranean Sea. This species is a good candidate for parentage analysis, as territorial males are confined to a very small area during the reproductive period. Recruiting juveniles are readily collected on scuba, which facilitates sibship analysis, and allows us to compare and demonstrate how these two relationship inference methods can be used to elucidate patterns of larval dispersal. We use the results of these analyses to provide the first direct estimates of the distribution of larval dispersal distances and directionality for a fish species in an open-coast temperate marine ecosystem.

## 2. Material and methods

### (a) Study species, study area and sampling

**Figure 1.**
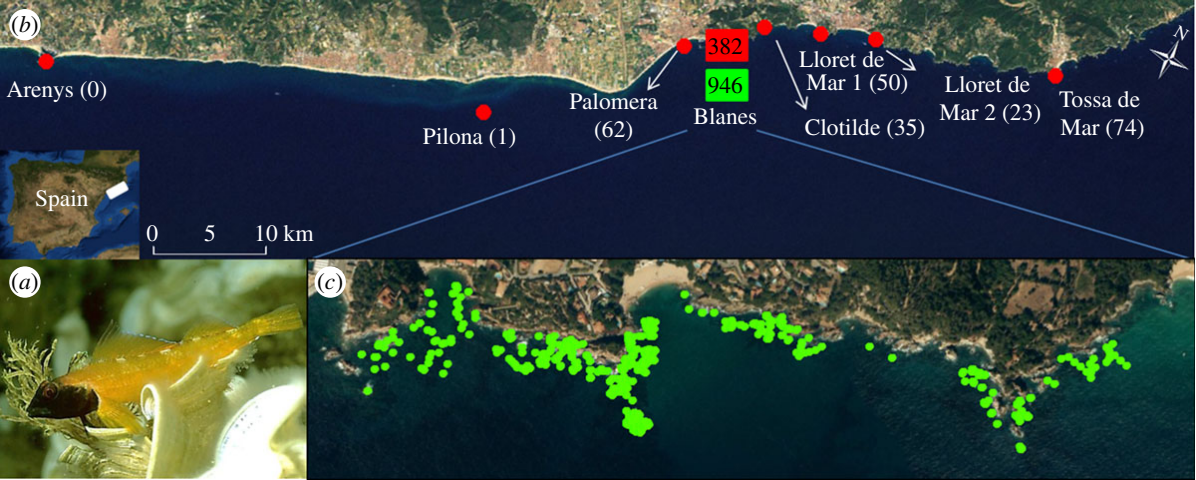
(*a*) Territorial male of *T. delaisi*. (*b*) Map of the sampling area with location names and number of juveniles sampled in brackets. Note that from Palomera towards the southwest, there are no rocky reefs apart from Pilona and Arenys. The squares represent the number of individuals analysed from the Blanes intensive sampling area, with adult sample size in light shade (online: green) and juvenile sample size in dark shade (online: red). (*c*) The Blanes intensive sampling area, where all dominant males encountered were sampled in their nests, indicated with light/green dots. (Online version in colour.)

The black-faced blenny (*T. delaisi*) is distributed throughout the Mediterranean Sea and the northeastern Atlantic Ocean [17] in near-shore rocky reef habitat, where they live camouflaged within the rocks or algae for most of the year. When the reproductive period starts, in spring, some males, known as territorial or dominant males, undergo coloration changes that result in a black head and bright yellow colouring for the rest of the body, distinguishing them from other members of the species (figure 1*a*). These males protect a small territory, which is referred to as their nest, against predators and uncoloured sneaker males [18]. The black-faced blenny also displays a high level of homing behaviour [19], and adults do not disperse even small distances in open water and sandy bottom habitat. Consequently, dispersal, as well as gene flow, is confined to the pelagic larval stage.

The study area is on an open Mediterranean coastline of northeastern Spain, near the port of Blanes (figure 1*b*). It is centred on a rocky shore of approximately 2 km in length, surrounded by sandy bottom habitat that extends for many kilometres in both directions, but especially towards the southwest (figure 1*c*), and could act as a dispersal barrier [20].

A total of 946 adult fish were sampled on scuba with fish nets between April and July 2010. Exhaustive searching for complete coverage of all nests in the 2 km section of coastline (Blanes area; figure 1*c*) was performed and 796 territorial males were sampled from their nesting sites. Additionally, 150 camouflaged adults of unknown sex were sampled. Fish were caught with small nets, body length measured and a small tissue sample taken from the dorsal fin before release.

A total of 627 juveniles were collected on scuba between the months of July and September 2010. Juveniles were sampled in the Blanes area, as well as at seven external locations, spanning a distance of 48.2 km, between Arenys and Tossa de Mar (figure 1*b*). Four of these locations are found to the northeast of Blanes, where, after a large sandy bottom stretch, the rocky shore continues providing habitat for *T. delaisi*. Southwest of Blanes, juveniles were sampled at all the available sites with rocky habitat (figure 1*b*).

### (b) Genotyping and genetic analyses

All 1573 samples were genotyped at 192 SNP markers (electronic supplementary material, table S1), developed for *T. delaisi*, using 96.96 Dynamic SNP Genotyping Array on an EP1 Genotyping System (Fluidigm Corporation) [21]. Deviations from Hardy–Weinberg (H–W) and linkage disequilibria were evaluated with Genepop v. 4.2 [22] and loci not in H–W equilibrium, as well as the less variable locus of pairs in linkage disequilibrium, were eliminated. Genotypes from the remaining 178 SNP loci were used for further analysis. Details of SNP development and of all genotyping assays are described by Schunter *et al.* [21].

Two kinship analyses were performed: parentage and sibship. Parent–offspring matches were established using the software CERVUS [23], which accommodates a large number of loci and has been shown to accurately identify parent–offspring pairs in empirical data [24]. The genotyping error rate was set to 1%, and a range of values between 25 and 95% for the parameter ‘adult proportion sampled’ was evaluated. A maximum of three mismatches was allowed. The critical LOD value was set at 95% confidence, but the final LOD value cut-off for identifying parent–offspring pairs was always more than or equal to 2. This is a conservative approach that accounts for some not-sampled putative parents in the population. Estimates of the probability of excluding a parent even when the parent was sampled (type I error) and of the probability of assigning a false parent whether the true parent was sampled or not (type II error) were evaluated with CERVUS. The program COLONY [25] was used to verify parent–offspring pairs identified with CERVUS.

COLONY [25] was also used to identify siblings in the juvenile samples. The predefined parameters were used, except that the mating system was set to polygamy. Full sibship was only accepted for posterior probability values of more than 0.75. The dataset was also permuted five times with sgm-perm [26] to obtain randomized datasets. The analyses were repeated five times with these permuted datasets to estimate the probability of assigning sibship pairs by chance alone. The program ML-RELATE [27] was also used to verify sibling assignments.

In order to identify any potential genetic structure within the samples, a Bayesian clustering analysis was performed with STRUCTURE v. 2.3.4 [28]. The analyses were carried out with all 1573 samples included, as well as with juveniles and adults separately. For each possible number of genetic units (*K* = 1–3), the values over 10 runs were averaged with 100 000 iterations discarded as burn-in and 200 000 iterations retained. Genetic structuring between juveniles from Blanes and the other sampling locations was evaluated with *F*_ST_ calculated using Genepop v. 4.2 [22]. Geographical distances between individuals were estimated from the sampling coordinates (electronic supplementary material, tables S2 and S3) with the R package *fields* [29].

### (c) Otolith analysis and wind variables

The body length of all juveniles was measured, and the lapilli otoliths were extracted and mounted on microscope slides. The age of individuals was determined in order to establish the corresponding dates of hatch and settlement, as well as pelagic larval duration (PLD), following Raventós & Macpherson [30].

The main documented superficial currents in this region of the Mediterranean Sea do not reach the coastal area, and therefore do not affect near shore species [31]. However, wind characteristics have a strong influence on the inshore circulation pattern in the study area [32,33]. As *Tripterygion* larvae are always distributed along inshore waters (less than 2.5 km from shore) [34], we compared the larval dispersal distance and direction with wind speed and direction to evaluate possible correlations with dispersal patterns. Wind data were obtained from an automated meteorological station belonging to the XMET service (National Weather Service of Catalonia). Wind data were recorded hourly with an anemometer 10 m above the ground. The overall wind regime in the study area revealed a clear diurnal pattern with stronger and more variable winds due to solar heating during the day, and mostly weaker and less variable winds blowing along the coast at night [33]. The wind data were therefore divided into day and night datasets by averaging the 12.00 and 00.00 measurements, respectively. The settlement date of each juvenile (as derived from otolith readings) and the corresponding PLD value were used to determine the period over which the wind variables were to be averaged. Day- and night-time daily averages were calculated, and wind speed and direction combined into a single coarse wind variable (electronic supplementary material, table S4) and compared with dispersal patterns using a Spearman's rank correlation test.

## 3. Results

**Figure 2.**
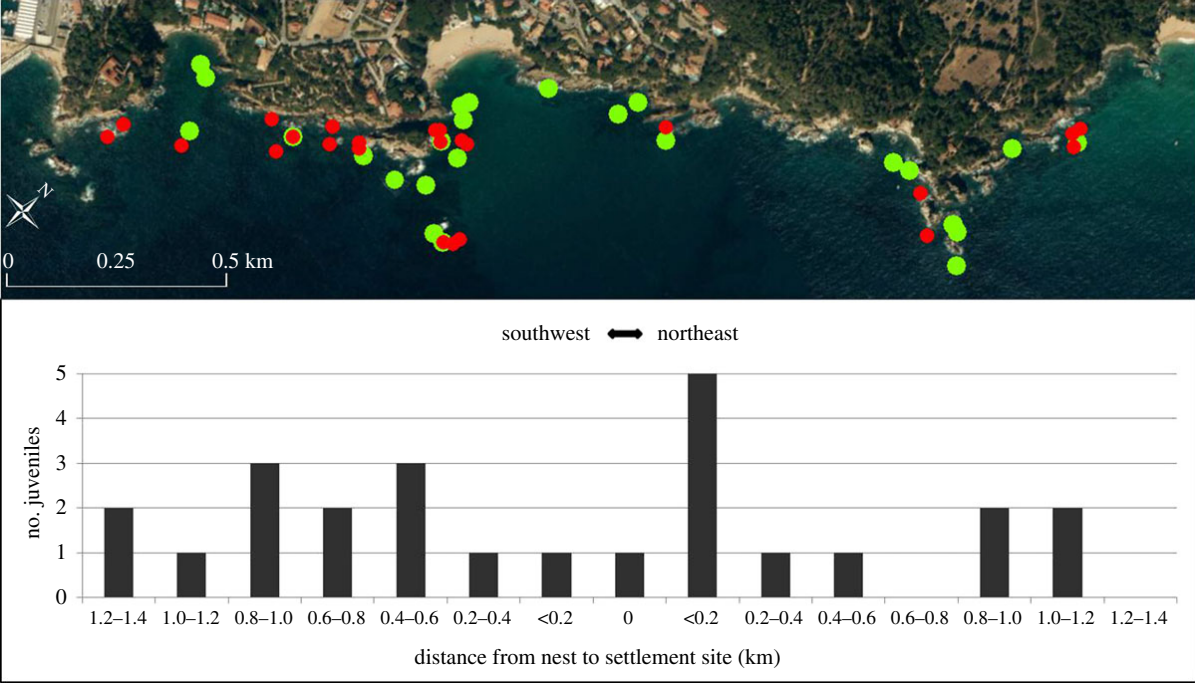
Map of the Blanes intensive sampling area with the location of the 25 identified parent–offspring pairs. The parent locations are marked in light shade (online: green) and the self-recruits locations in dark shade (online: red; two self-recruits were found on the exact same rock, so the map has 24 dark/red dots). The histogram represents the movement of self-recruits from their natal nest to their recruitment site as the shortest Euclidian distance. The right side of ‘0’ represents the self-recruits that moved towards the northeast and the left side those that moved towards the southwest. (Online version in colour.)

**Figure 3.**
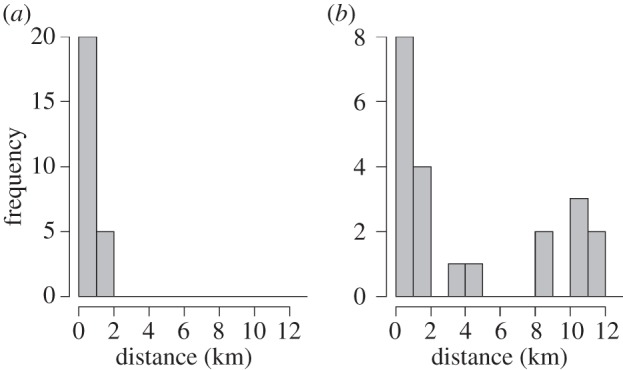
Geographical distance of dispersal between (*a*) parent–offspring and (*b*) full sibling pairs.

A total of 25 parent–offspring pairs were identified, all in the Blanes area (figure 2). The same 25 pairs were found with both parentage methods, CERVUS and COLONY. Using the 95% critical LOD value given by CERVUS, the number of parent–offspring pairs varied from 25 (25% of adults sampled) to 119 (95% of adults sampled). By using a conservative threshold of LOD more than or equal to 2, which was the 95% critical LOD for 25% of adults sampled, the same 25 pairs were detected independent of CERVUS input parameters. Type I error estimates were all 0 and the type II error varied from 0 to 0.0041 (25% and 95% adults sampled, respectively). We found that 6.5% of the juveniles from the Blanes area were self-recruits (figure 2), with one of these offspring found directly adjacent to the fathers’ nest. Self-recruits did not disperse further than 1.2 km within the 2 km intensive sampling area, with 80% settling less than 1 km from their natal nest (figure 3).

Sibship analysis with COLONY identified 21 pairs of juveniles as full siblings. ML-RELATE identified six of these 21 pairs as full siblings, whereas the other 15 pairs had similar likelihoods of being full or half siblings. No parents were identified for any of the 42 juveniles in these pairs. By permuting the juvenile dataset, we found that the probability of assignments due to chance is low (0.0028%). As *T. delaisi* adults do not disperse and males territorially guard one nest, even half siblings will come from either the same nest (paternal) or close nests (maternal). In either case, these 21 identified pairs are clearly siblings and can be assumed to have dispersed from approximately the same location. The geographical distances between siblings were larger than those between parent–offspring pairs. The maximum distance between siblings was 11.5 km, with 24% of sibling pairs separated by more than 10 km and 38% dispersing less than 1 km from each other (figure 3). Even if the siblings had dispersed in opposite directions from their natal nest, meaning that we observe the largest possible dispersal distance, their expected trajectory is larger than the average dispersal trajectory identified by parentage analysis.

We found no population structure within *T. delaisi* from our study area, as the STRUCTURE analysis identified *K* = 1 to be the most probable number of genetic clusters (electronic supplementary material, figure S1) when combining all samples. The same result was encountered when adults and juveniles were analysed separately (data not shown). Furthermore, the *F*_ST_ estimate between juveniles collected in Blanes and those from the other sampling locations was low (*F*_ST_ = 0.0005) and not significantly different from zero.

Dispersal distance did not correlate with the wind variable, which included both wind direction and strength, and was calculated for both day- and night-time (day component versus distance: Spearman's *ρ* = 0.265, *p* = 0.22; night component versus distance: Spearman's *ρ* = −0.177, *p* = 0.42). The average day-time wind component was −1.675 ± 0.175 and the average wind direction was 170 ± 3.9, which means the wind blows away from the coast during the day. The average wind component at night was 0.411 ± 0.128 and the average wind direction was 260 ± 9.3, which means the wind blows in a southwesterly direction at night. When comparing the wind component with the dispersal direction of self-recruits, we found no correlation with the day-time wind component, but a nearly significant correlation at night (day component versus direction: Spearman's *ρ* = 0.179, *p* = 0.41; night component versus direction: Spearman's *ρ* = −0.400, *p* = 0.06). There were 13 self-recruits that moved in a southwesterly direction from their natal nest and 11 self-recruits that dispersed towards the northeast. One recruit was found on the same rock as its parental nest. The dispersal direction was highly correlated with the location of the parental nest within the sampling area (Spearman's *ρ*: *r* = −0.710744, *p* > 0.0001). We find northeastern dispersal to be of shorter distance (figure 2), while not statistically significant (Mann–Whitney *U*-test, *Z* = −1,18770, *p* = 0.234953). Furthermore, no juvenile collected northeast of Blanes was an offspring of any of the fathers in the Blanes area, which suggests that few larvae disperse great distances to the northeast.

## 4. Discussion

Here, we demonstrate how two genetic techniques for determining kin relationships, parentage and sibship reconstruction analyses, can be used to elucidate fine-scale patterns of larval dispersal for a rocky reef fish in an open-coast temperate ecosystem. Self-recruitment, which is the percentage of larvae that settle back to the natal location, in marine fishes has long been debated [35], but empirical studies over the last 15 years have begun to elucidate patterns [35,36]. The degree to which a population receives self-recruits has ecological implications, as it influences the level of self-replenishment and population sustainability [37]. Studies using parentage analysis to study dispersal in several species of coral reef fishes inhabiting embayments in Papua New Guinea revealed high self-recruitment [4,7], with 40–64% of juveniles recruiting to their natal site. Studies using a similar approach with coral reef fishes but on open coastlines found the proportion of self-recruiting juveniles to average 4.6–7.5% per site [11,38]. The percentage of self-recruitment for *T. delaisi* on a temperate open coastline found here (6.5%) was similar. Saenz-Agudelo *et al*. [11] argue that coastal geography may be crucial in explaining the relatively low rates of self-recruitment. It has been suggested that habitat patchiness determines the openness of populations in marine species with different dispersal abilities [39]. Temperate open coastlines typically lack a network of suitable habitat, as is often found in a tropical reef environment, and recruitment is only possible on a narrow strip of rocky shoreline habitat. This suggests that the proportion of self-recruiting individuals is reflected by the openness or patchiness of the habitat geography, or lack of available habitat.

Pelagic larval duration has been suggested to have a large influence on the rate of self-recruitment [5,29]. However, this does not seem to be the case for *T. delaisi*, as we detected a low proportion of self-recruits, with an average PLD of 18 days, and a similar proportion was estimated for a coral reef species with a PLD of just several days [6]. Nevertheless, the percentage of self-recruitment found in *T. delaisi* is a minimum estimate, because sneaker males may also contribute to the progeny. We sampled all the territorial males observed in the study area and tried to capture sneaker males, but were largely unsuccessful, as they are camouflaged and quite difficult to see. However, sneaker males are rarely observed to participate in mating, suggesting generally low reproductive output for this behavioural strategy [40] and a minor contribution to the proportion of self-recruits in this population.

Large-scale investigations that integrate population genetics and oceanographic modelling have shown that connectivity can be influenced, or even dominated, by physical processes [41–44]. However, near-shore oceanographic features are generally hard to model, because they are mostly influenced by wind and coastal morphology [32]. For example, dispersal patterns of the coral reef fish *Amphyprion polymnus* were found not to be influenced by physical processes, such as currents [11]. For *T. delaisi*, we found no significant correlation between the dispersal of self-recruits and the component wind variable, probably due to the lack of a pattern of directional dispersal. None of the juveniles collected in the sampling areas northeast of Blanes were offspring of the Blanes parents, which could be due to the large population size. For those recruits that dispersed southwestward, the coastline is formed by sandy beaches with no available rocky reef habitat for approximately 100 km, suggesting little successful dispersal occurring beyond this area. It was previously reported that the population of Blanes is genetically distinct from that of the next analysed locality approximately 250 km to the southwest, with a low number of juveniles assigned to distant locations [17]. However, these populations are not completely disconnected, as isolation by distance was found along the Spanish coast [45], suggesting that a small number of larval recruits must successfully disperse long distances to the southwest. As such, the majority of larvae from this open-coastline habitat settle in suitable habitat adjacent to their natal location, but small numbers of larvae disperse further and allow large-scale connectivity.

Parentage analysis in *T. delaisi* revealed limited successful dispersal and only short-distance dispersal events in the focal study area. Identified dispersal events declined precipitously more than 1 km from the natal site, as previously found in reef fish species [8,38]. The low level of self-recruitment, though, would indicate high influx of recruits from other areas, which in turn suggests a larger dispersal range. Indeed, the *T. delaisi* population in the study area was found to be one genetic unit connected by high gene flow, consistent with previous studies [17,45].

The high connectivity found in population structure analyses was reflected in the sibling pairs identified. Sibship analysis has previously been used to successfully elucidate family structure and mating strategies in several other fish species [46–48]. Here, we use it to provide information on the dispersal potential of a species in the marine environment. Whereas paternity analysis can provide information on both dispersal distance and directionality, identification of siblings recruiting to different locations provides estimates of a possible range of dispersal distances, because full siblings begin dispersal from the same natal nest. The mean distance between *T. delaisi* sibling pairs was much larger than the maximum distance between parent–offspring pairs (figure 3). Nonetheless, sibship analysis could still underestimate dispersal distance, because the natal location is not known. If siblings disperse in opposite directions, then the distance between them will be twice their mean dispersal distance, but if they do not, then the observed distance will be less than their combined dispersal distance.

Combining kin relationship estimation with otolith analysis, it is also possible to retrace whether siblings recruited together. Indeed, a recent study on a coral reef fish provided evidence that siblings can travel together throughout the entire pelagic larval phase [49]. Nonetheless, there was only one *T. delaisi* sibling pair encountered at the same recruitment location. Otolith analysis found that both siblings hatched within 2 days of each other and spent 19 days in the pelagic larval phase, indicating that they probably left the nest at the same time and recruited together. Even though many groups of juveniles were collected simultaneously from the same locations, only this one pair of siblings was identified as having recruited together. This indicates that the majority of full siblings do not recruit to the same location, suggesting a high degree of larval mixing and recruitment heterogeneity [49].

In this study, by selecting to work with a species for which parentage analysis is possible due to the sedentary, territorial behaviour of adults, we can compare the results of the two kinship methods. With parentage analysis, the direction of dispersal events is known, which cannot be accomplished with sibship reconstruction as the location of origin of the siblings is generally not known. Nonetheless, in our study there was no clear pattern of dispersal direction, even though parentage analysis was performed. With sibship reconstruction, which does not require sedentary life history or sampling of parents, the dispersal range of the species is estimated, and inference regarding family structure and dispersal behaviour can be derived. For *T. delaisi*, the parent–offspring comparisons indicated that larvae tend to move short distances along the coast. By contrast, the sibling pairs identified showed that recruitment can be at locations quite distant from their natal site, and from each other, reflecting the high degree of connectivity inferred from population structure analyses.

The information provided by these two methods can be complementary, and provides a more complete picture of dispersal dynamics and population connectivity than either one alone. The direct measurement of dispersal distance and direction by applying parentage analysis is dramatically increasing our knowledge of larval dispersal in marine organisms. However, for many species, including those of conservation concern, sampling a large proportion of the parental population is difficult or unfeasible, or the life history may make it difficult to infer the origin of dispersal events. In such cases, sibship reconstruction can be more easily applied, as only one generation needs be sampled, to elucidate dispersal patterns and structure in marine species.

## Notes

Collection and field procedures followed Spanish Law (Royal Executive Order, 53/2013) for Animal Experimentation, in accordance with European Union directive 2010/63/UE.

## Data accessibility

Genotypes of *T. delaisi* adults and recruits at the 178 SNP markers can be found in the electronic supplementary material, table S1. Coordinates for each collected individual sample are in the electronic supplementary material, tables S2 and S3, and wind variables including wind direction and strength can be found in the electronic supplementary material, table S4.
